# Orchidaceae-Derived Anticancer Agents: A Review

**DOI:** 10.3390/cancers14030754

**Published:** 2022-01-31

**Authors:** Tomasz Śliwiński, Tomasz Kowalczyk, Przemysław Sitarek, Marta Kolanowska

**Affiliations:** 1 Laboratory of Medical Genetics, Faculty of Biology and Environmental Protection, University of Lodz, 90-236 Lodz, Poland; tomasz.sliwinski@biol.uni.lodz.pl; 2 Department of Molecular Biotechnology and Genetics, University of Lodz, Banacha 12/16, 90-237 Lodz, Poland; tomasz.kowalczyk@biol.uni.lodz.pl; 3 Department of Biology and Pharmaceutical Botany, Medical University of Lodz, Muszynskiego 1, 90-151 Lodz, Poland; przemyslaw.sitarek@umed.lodz.pl; 4 Department of Geobotany and Plant Ecology, Faculty of Biology and Environmental Protection, University of Lodz, Banacha 12/16, 90-237 Lodz, Poland; 5 Department of Biodiversity Research, Global Change Research Institute AS CR, Bělidla 986/4a, 603 00 Brno, Czech Republic

**Keywords:** orchids, secondary metabolites, anticancer

## Abstract

**Simple Summary:**

Orchids are commonly used in folk medicine for the treatment of infections and tumors but little is known about the actual chemical composition of these plants and their anticancer properties. In this paper, the most recent literature on orchid-derived bioactive substances with anticancer properties is reviewed. According to the published data, numerous species of orchids contain potential antitumor chemicals. Still, a relatively insignificant number of species of orchids have been tested for their bioactive properties and most of those studies were on Asian taxa. Broader research, ’including American and African species, as well as the correct identification of samples, is essential for evaluating the usefulness of orchids as a plant family with huge anticancer potential.

**Abstract:**

Species of orchids, which belong to the largest family of flowering plants, are commonly used in folk medicine for the treatment of infections and tumors. However, little is known about the actual chemical composition of these plants and their anticancer properties. In this paper, the most recent literature on orchid-derived bioactive substances with anticancer properties is reviewed. For the assessment, previous papers on the anticancer activity of Orchidaceae published since 2015 were considered. The papers were found by exploring electronic databases. According to the available data, many species of orchids contain potential antitumor chemicals. The bioactive substances in a relatively insignificant number of orchids are identified, and most studies are on Asian taxa. Broader research on American and African species and the correct identification of samples included in the experiments are essential for evaluating the usefulness of orchids as a plant family with vast anticancer potential.

## 1. Introduction

According to the World Health Organization (WHO) “Guidelines on Safety Monitoring of Herbal Medicines in Pharmacovigilance Systems”, up to 80% of the world’s population rely on herbal medicines as a primary source of healthcare. As summarized by Ekor [1], the use of herbal medicines is increasing also in developed countries [2,3]. It is not surprising that the utilization of plants in medicine is as old as mankind itself and even modern pharmacotherapy includes numerous herb-derived drugs [4,5]. Noteworthy, about 10% of known vascular plants are currently used as therapeutics [6]. In 2019 alone, almost 2000 new species of plants were discovered according to the “State of the World’s Plants and Fungi 2020” report, published by the Royal Botanic Gardens Kew. These plants could be potential sources of new phytochemicals that can be used in medicine [7].

Cancer ranks as a leading cause of premature death around the globe. In 2020, 19.3 million new cases of cancer cases and almost 10 million cancer deaths were reported worldwide [8]. The most common were female breast tumor (11.7%), lung cancer (11.4%), and colorectal cancer (10.0%) [8]. Moreover, about a 47% increase in the yearly diagnosed cancer cases is expected to occur in 2040 [8]. Cancer treatments include various medical procedures, e.g., surgical treatment, radiotherapy, and chemotherapy. However, conventional chemotherapeutic agents can fail as a result of chemoresistance development [9,10] and about 80–90% of the deaths from cancer are assigned to this resistance [11,12]. Natural products have the potential for overcoming drug resistance [13,14,15,16]. Obviously, plants serve as an important source of useful anticancer bioactive compounds and previous records indicated that about 60% of the currently used antitumor drugs are derived from natural products [17,18,19,20]. 

The aim of the present study is to summarize recent advances in research on orchid metabolites with anticancer properties.

## 2. Criteria for Selection of Experimental Papers 

This review includes papers regarding the anticancer activity of orchid compounds published since 2015. The papers were found using electronic databases PubMed/MEDLINE, Scopus, Web of Science, and Google Scholar. The quest terms included Orchidaceae alone and as well as with the following: plant extract, derived compounds, tumor, cancer, lung cancer cells, colon cancer cells, breast cancer cells, prostate cancer cells. Experiments on extracts and secondary metabolites of orchids with in vitro activity against various cancer cell lines were also included in the review. Research published in languages other than English and Spanish or without abstracts in these languages, without full access to the complete text, lacking the identification of the taxon at the species level or without a clear objective and methodology were not analyzed. The removal of duplicates of articles obtained from the electronic databases was followed by the verification of other criteria listed above.

## 3. Orchidaceae

Orchidaceae is one of the largest families of flowering plants with more than 27,000 accepted species [21] and more than 31,000–35,000 species are estimated to exist in total [22,23]. This is a cosmopolitan group growing in almost every habitat except deserts and glaciers. The plants are found above the Arctic Circle, in Patagonia, and even on Macquarie Island [24,25]. However, the greatest diversity of orchids is recorded in tropical regions, especially in mountainous areas [26].

Orchids can grow as epiphytes, lithophytes, or terrestrial perennial herbaceous plants that lack any permanent woody structures. Adult plants are mostly able to acquire carbon through photosynthesis, but some taxa are mycoheterotrophic [27]. Orchids are extremely diverse and their growth can be sympodial or monopodial. Many species produce storage organs like bulbs or pseudobulbs. Their flat or pleated leaves may be variously arranged on the stem (alternate, opposite, arranged spirally), or may grow only at the base of the plant. Orchid flowers are extremely diverse, usually zygomorphic, and most often containing both male and female reproductive organs. The outer whorl has three sepals and the inner whorl has three petals; however, one petal (lip) is usually modified and differs in appearance from the other two. A central flower structure called the column comprises both the male (anther) and female (stigma) parts of the flower. The ovary is composed of three carpels. 

Orchids are often called “masters of deception” due to the diversity of deceptive mechanisms for attracting pollinators, e.g., generalized food deception, food-deceptive floral mimicry, brood-site imitation, shelter imitation, pseudo antagonism, rendezvous attraction, and sexual deception [28,29]. Many nectar-less orchids mimic other pollinator-rewarding plants [30] or produce various pseudo pollen or pollen-like papillae to lure insects [31]. A large group of species is able even to produce chemicals similar to insect sex pheromones [32,33,34,35] and this means of pollination, called pseudo copulation, is found only in orchids.

Noteworthy, throughout their life orchids, are associated with mycorrhizal and non-mycorrhizal fungi [36,37,38,39,40]. These endophytes most probably increase or modify the production of plant secondary metabolites [40,41,42,43,44]. 

Orchids became one of the most popular ornamental plants in the Victorian era and currently, the official global orchid trade is estimated to ca. 72 million specimens per year [45]. These plants are widely used as medicines, food, and as herbs with other cultural values [46,47]. Currently vanilla together with salep and chikanda are globally and regionally important food products [45]. Orchids were first used in Chinese traditional medicine [48,49], but they are also popular in Ayurvedic therapies [50] and are commonly used by native tribes in tropical America as well as in Africa [51,52].

Noteworthy, despite a long history of orchid research, scientists are still finding many new species in the tropical areas e.g., [53,54,55]—only in 2020 more than 100 orchids were described, most of them from South America [56,57,58,59,60,61,62,63] and tropical Asia [64,65,66,67,68,69]. Unfortunately, the taxonomists did not analyze the chemical properties of their secondary metabolites and their potential medicinal usefulness remains unrecognized. 

### 3.1. Importance of Symbiosis

As mentioned before, all orchids are associated with specific mycobiota and different fungi species are found in various plant parts [40,70,71,72,73]. Preliminary studies already proved that some of these microorganisms are characterized by antimicrobial activities [74,75] and that interactions of symbiotic fungi with plants contribute to secondary metabolites production .

Unfortunately, the comparative studies on compounds extracted from fungi-infected and *in vitro* cultivated, fungi-free orchids were not conducted so far. Considering the enormous number of orchid species, their symbionts remain poorly recognized. Noteworthy, most of the experiments on orchid endophytic fungi included only root tissue, [76,77] while in traditional medicine, stems, and leaves are organs usually used for therapies [43,48,49,78,79,80,81]. The importance of recognition of orchid endophytic fungi for secondary metabolites synthesis and their potential application in medicine were summarized by Sarsaiya et al. [39] and Pant et al. [82].

Interestingly, some of the bioactive compounds were found in an invasive orchid species, *Arundina graminifolia*, which is an Asian native herb. It would be important to study also the populations of this species which are currently invading Central and South America [83] in the context of the differences in symbiotic mycobiota of non-native plants as well as the similarity of secondary metabolites produced by native and invasive populations. Similarly, the compounds produced by *Liparis nervosa* which grows in Asia, Africa, and America should be compared with plants collected in various geographical regions.

### 3.2. Importance of Taxonomy and Plant Material Preservation

In this study, as experts in orchid taxonomy [84,85,86,87], it is crucial to emphasize the fundamental role of the correct identification of plants for further studies on the usefulness of phytochemicals in cancer therapy [88,89,90]. The diversity of orchids and superficial similarity of related species often leads to erroneous identification of taxa [91,92]. The detailed studies on various orchids revealed that numerous commonly recognized species are actually species-complexes that include several distinct species [93,94,95].

Most of the reports reviewed in this paper were on *Dendrobium,* which is one of the most complicated taxa in terms of species nomenclature and classification [96]. Currently, there are more than 1000 species in this genus, and new species and varieties are described frequently from tropical Asia [97,98]. Diagnostic characters which allow to identify particular *Dendrobium* species are related to flower morphology and therefore plants cannot be correctly classified in the vegetative stage [99]. To further investigate orchids used for treating cancer, it is vitally important that they are correctly identified. Initial identification of a plant should not only be confirmed by expert taxonomists but also voucher material further verified and preserved in the form of dried herbarium specimens [100] and preferably complemented with DNA barcodes [101,102,103]. The molecular identification without properly preserved plant material can be doubtful [104,105]. Unfortunately, the good practices summarized by Bussmann [91] are rarely applied in studies on orchid secondary metabolites, therefore it is not possible to confirm the identification of examined species.

## 4. Secondary Metabolites of Orchids

The basic knowledge on the diversity of orchid secondary metabolites was summarized by Sut et al. [71], Teoh [106], and Pant et al. [82] but the authors of these papers did not present data on the action mechanism of particular secondary metabolites, the importance of symbiotic fungi or other issues related with using orchid-derived biocompounds. Experiments on alkaloids, terpenes, stilbenoids, bibenzyls, phenanthrenes, flavonoids, and polysaccharides isolated from Orchidaceae indicated their potential medical usefulness [106]. 

Gigantol and batatasin III are the main bibenzyls occurring in orchids with cytotoxic activity [107,108]. Phenanthrenes are common metabolites of orchids used in traditional medicine [106]. Many of them are cytotoxic and kill human cancer cell lines and possess antiallergic, antimicrobial, antiinflammatory, antioxidant, antiplatelet, and spasmolytic properties [109]. Antitumour properties are reported for monomeric phenanthrenes, biphenanthrenes, and triphenanthrenes [106]. It is also reported that phenanthroquinone (denbinobin) and dihydroxymethoxy phenanthrene (lusianthridin) are cytotoxic [110,111,112]. Bibenzyl derivatives of phenanthrenes are effective anti-tumor chemicals [113,114,115]. Alkaloids are another group commonly isolated from orchidis that are important in a medicinal context [48]. Orchid alkaloids are usually classified either as the pyrrolizidine type or the dendrobine-type [106]. Denbinobin triggers apoptosis of numerous human cancer cell lines [70,109,116,117]. A large number of compounds, estimated to exceed 10 000, are aromatics flavonoids, phenols, and tannins [71]. These chemicals have a broad range of pharmacological activities that involve i.a. antioxidant, antimicrobial, antiinflammatory, antimutagenic, antitumour, and immuno-modulatory activities [106,118,119,120]. 

Noteworthy, some of the bioactive compounds can be actually produced by the symbiotic microbes associated with orchids [121]. 

It should be emphasized that our team is also currently working on the identification of phytochemicals in the orchid species described for the first time and on the determination of their biological properties, including anticancer activity.

## 5. Biotechnological Methods for Orchidaceae Family

As shown, plants of the Orchidaceae family can be a source of many valuable, biologically active compounds that can be widely used as a basis or a supplement to the modern forms of oncological therapy. Plants growing in natural habitats are often the only source of these valuable compounds. Due to the fact that these plants usually do not synthesize large amounts of these compounds, it is very difficult to meet the constantly growing demand for these metabolites. What is more, many species capable of their synthesis are under strict protection. The solution to this problem is the use of biotechnological methods allowing constant access to valuable biomass from in vitro cultivation and, in many cases, increasing the level of their synthesis and accumulation. For this purpose, efficient in vitro propagation protocols have already been developed for many medically valuable orchid species. Such an approach often involves the induction of callus tissue which can then be stimulated to differentiate to give rise to new shoots, or in the case of embryogenic callus, it may be the start of somatic embryos. Pujari et al. described three simple, fast, and economical in vitro tissue culture protocols for Dendrobium ovatum that can be used to develop the right amount of material for biological research in an endangered orchid. Additionally, the authors also demonstrated the enhancement of moscatilin production in the in vitro cultures of this valuable plant [122]. Another type of culture that has found application for the Orchidaceae is the protoplast or thin cell layer (TCL) culture. Vudala et al. developed an effective micropropagation protocol for Hadrolaelia grandis with thin cell layer culture systems that can be the starting point for in vitro plant breeding, even on a large scale [123]. Additionally, Brattacharyya et al. developed a protocol for the regeneration of *Dendrobium aphyllum*, an important therapeutic orchid by the t-TCL method. For this purpose, Murashige and Skooga (MS) medium was supplemented with 15 µM meta-topoline along with 10 µM TDZ and 10 µM AgNO_3_. This combination was found to be the most optimal for shoot proliferation [124]. In addition, an adventitious shoot can also be a valuable strategy, which in a relatively short time, using appropriate growth regulators, allows to multiply valuable plant material. As presented by Mahendran et al. who developed a protocol for induction of direct somatic embryogenesis and subsequent plant regeneration for the medicinally important and endangered plant of Malaxis densiflora. In these in vitro studies, seed-derived protocorm explants were cultured on 1/2 Murashige and Skoog medium with 2,4-D, Picloram, and Dicamba alone or in combination with BAP, TDZ, and Kn. It was shown that the best results were obtained on 1/2 MS with 3.39 μM of 2,4-D and 6.80 μM of TDZ. This protocol is another example of work on the possibility of efficient in vitro culture of human-important members of the Orchidaceae family [125]. Another strategy worth considering, among the sources of extremely valuable compounds, is the cultivation of various tissue and cell cultures in special bioreactors [126,127]. These devices, which allow for the maintenance of plant material in sterile conditions in vitro, often allow the optimization of the entire breeding process, which is extremely important from a technological and economic point of view. Bioreactors ensuring optimal conditions for growth and development by strict control of many key parameters have long been used even on an industrial scale in many other plant families. In addition, the possibility of stimulating production with various physical and chemical factors, combined with genetic modifications in the future, will certainly allow the development of efficient and comprehensive solutions allowing the use of the Orchidaceae family as a kind of mini-factories producing compounds desired in many areas of life.

## 6. The Anticancer Activity of Plant Extracts from Orchidaceae

Extracts of many species of orchids have anticancer properties. Isolates from various plant parts exhibit cytotoxic activity against leukemia and melanoma, as well as against brain, breast, cervical, gastric, liver, and lung cancer cells.

**Figure 1:**
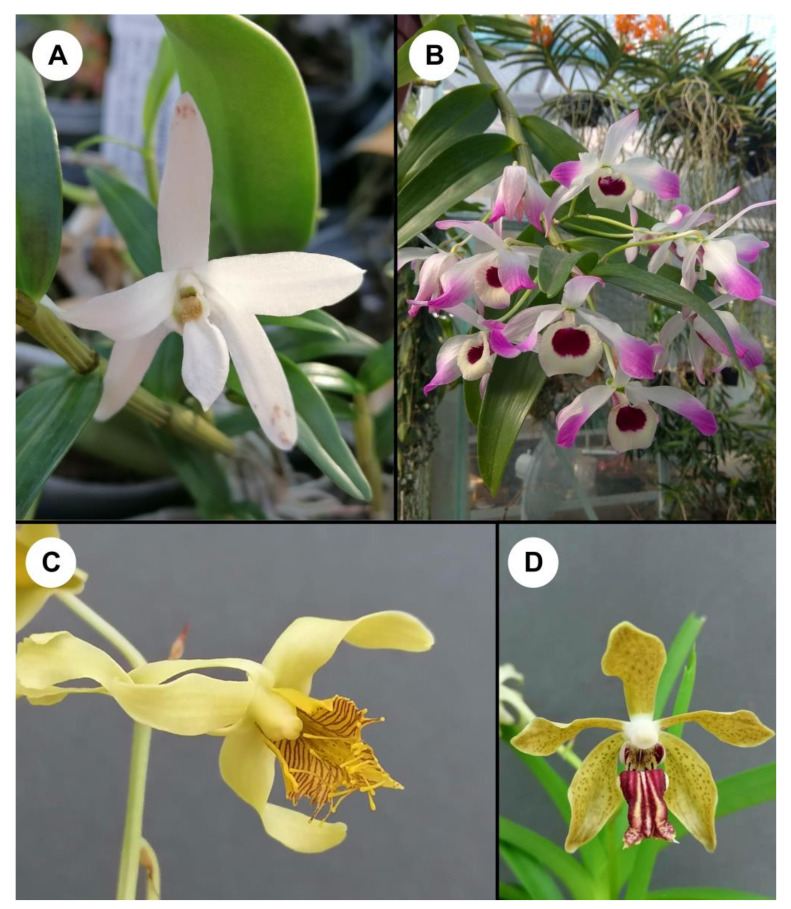
Some orchids with bioactive, antitumor compounds. (**A**)—*Dendrobium moniliforme*, (**B**)—*Dendrobium nobile*, (**C**)—*Dendrobium venustum*, (**D**)—*Vanda cristata*

Extracts of several species of *Dendrobium* (Figure 1) have a cytotoxic effect and inhibit the growth of cervical cancer and glioblastoma brain tumor cells [128,129,130,131,132]. It is hypothesized that polyphenol compounds found in orchid extracts inhibit cancer cells by xenobiotic-metabolizing enzymes altering the metabolic activation of potential carcinogens [133]. On the other hand, flavonoids can modify hormone production and prevent the growth of cancer cells [133]. In contrast, phenolics can interrupt cellular division during the telophase stage of mitosis. These chemicals also affect cell proliferation by reducing the amount of cellular protein, the mitotic index, and colony formation [131]. The ethanolic extract of *Dendrobium chrysanthum* perturbs cell cycle progression and results in a delay in the growth of cells. It also exerts anticancer activity [129]. A similar situation for extracts of *D. venustum* in which phoyunnanin E triggered apoptosis of lung cancer cells by suppression of survivin [134]. Another *Dendrobium* species, *D. crepidatum*, is significantly cytotoxic against both cervical cancer (HeLa) and glioblastoma brain tumor (U251) cell lines [128].

Joshi et al. [130] indicate that *Vanda cristata* (Figure 1) is both cytotoxic against cervical cancer (HeLa) and glioblastoma brain tumor (U251) cell lines, while *Vanda cristata*, *Pholidota articulate, and Papillionanthe uniflora* exhibited significant cytotoxic activity against cervical cancer (HeLa) cells.

Another promising genus with anticancer properties is *Bulbophyllum. B. kwangtungense*, and shows antitumour activity against cervical cancer (HeLa) and leukemia (K562) cell lines [130,135]. *Bulbophyllum odoratissimum* is also cytotoxic against leukemia cell lines (K562, HL-60), hepatoma (BEL-7402), lung adenocarcinoma (A549), and stomach cancer (SGC-7901) cell lines [136]. Extracts of *Bulbophyllum sterile* bulbs and roots cause apoptosis in human colon cancer (HCT116) cell lines by arresting the G2/M phase of the cell cycle [137].

The volatile oil of *Anoectochilus roxburghii* induces apoptosis in tumor cells and triggers an enzyme cascade resulting in the apoptosis of lung cancer cells (NCI-H446) [138]. The ethyl acetate extract of *Anoectochilus formosanus* induces apoptosis in human breast cancer cells (MCF-7) and the aqueous extract effectively inhibits the growth of colon cancer cells in mice [138].

Some studies on *Pleione* by Liu et al. [139] indicate that an ethyl acetate extract of *Pleione bulbocodiodes* inhibits the growth of mice cancer cells LA795 (lung adenocarcinoma). Wang et al. [140] also indicate that some components of the extract of *Pleione yunnanensis* strongly inhibit the growth of lung adenocarcinoma cells. Other compounds obtained from this species are very cytotoxic against colon cancer cells (HepG2), liver cancer cells (BGC-823), and breast cancer cells (MCF-7). 

Other orchid extracts that are effective against breast cancer (MCF-7) are those from *Eulophia nuda* tubers [141], leaves of *Aerides odorata* [142], and leaves of *Vanilla* [143]. It is hypothesized that the cytotoxic activity is related to the synergistic action of the phytoconstituents present in these species [141]. Other studies are presented in Table 1.

## 7. The Anticancer Effect—Potential Mechanism of Action and Activation of Signalling Pathways of Pure Compounds from Orchids

Several classes of phytoconstituents of great chemical diversity have been isolated from therapeutically-used orchids [71].

Various stilbene-based derivatives from orchids, e.g., pholidonone [158], bletilols [159] are cytotoxic against cancer cell lines. The former compound triggers apoptotic cell death in human gastric cancer cells, by inducing ER stress, probably via PERK and IRE1α signalling pathways [158].

Another group of orchid metabolites that have antitumor activities are phenanthropyrans and phenanthrenes [160,161,162,163,164,165,166]. Nudol isolated from *Dendrobium nobile* arrests the cell cycle of osteosarcoma (U2OS) cells, induces cell apoptosis via the caspase-dependent pathway and suppresses the migration of these cells [161]. Cypripedin isolated from *Dendrobium densiflorum* is effective against lung cancer by activating caspase-3 and downregulating the antiapoptotic proteins Bcl-2 and Bcl-xL in cells [167]. Denbinobin also isolated from *Dendrobium* and *Ephemerantha* also promotes caspase-3 activity in lung adenocarcinoma cells [168,169] and a polysaccharide extracted from *Anoectochilus roxburghii* inhibits in this way the growth and proliferation of human prostate cancer (PC-3) cells [138]. 

Spiranthesphenanthrene isolated from *Spiranthes sinensis* is cytotoxic against gastric cancer (SGC-7901), hepatocellular carcinoma (HepG2), and melanoma tumor (B16−F10) cell lines [170]. Moreover, this compound significantly inhibits the migration of melanoma tumor (B16−F10) cancer cells [170]. 

Bulbocodioidins extracted from *Pleione bulbocodiodes,* which are phenanthrene and phenanthrene/bibenzyl atropisomers, and according to Wang et al. [171], are cytotoxic activity against colon cancer (HCT-116), liver cancer (HepG2), and breast cancer (MCF-7) cell lines. Previously the bibenzyls isolated from this plant were shown to significantly inhibit the growth of leukemia cells (K562, HL-60), liver cancer cells (BEL-7402), gastric cancer cells (SGC-7901), lung cancer cells (A569, H460), and melanoma cells (M14).

Isoviolanthin isolated from *Dendrobium officinale* reverses TGF-β1-mediated epithelial-mesenchymal transition in hepatocellular carcinoma (HCC) cells by deactivating the TGF-β/Smad and PI3K/Akt/mTOR signalling pathways [172].

Phenanthrene and bibenzyl derivatives isolated from *Cremastra appendiculata* are cytotoxic against colon cancer (HCT-116), liver cancer (HepG2), stomach cancer (BGC-823), lung cancer (A549), and glioma cancer (U251) cell lines [163,173].

One of the most extensively studied orchid compounds is dendrobine and its derivatives [174,175]. This chemical induces apoptotic cell death via a mitochondrial-mediated pathway in lung cancer cells (A549). The combination of dendrobine with cisplatin enhances their cytotoxicity by stimulating JNK/p38 stress signalling pathways and, consequently, inducing apoptosis involving the pro-apoptotic proteins Bax and Bim [176]. 

ViceninII, which is flavonoid glycoside extracted from *Dendrobium officinale,* inhibits transforming growth factor-β1 (TGF-β1)-induced epithelial-mesenchymal transition (EMT) by deactivating TGF-β/Smad and PI3K/Akt/mTOR signalling pathways in lung adenocarcinoma A549 and H1299 cells [177].

Recent experiments indicate that erianin isolated from *Dendrobium* induces ferroptotic cell death in lung cancer cells (H460 and H1299). This action is accompanied by ROS accumulation, lipid peroxidation, and GSH depletion [178,179]. Other research examples are presented in Table 2. 

## 8. In Vivo Studies of Extracts and Pure Compounds from the Orchidaceae Family

**Figure 2:**
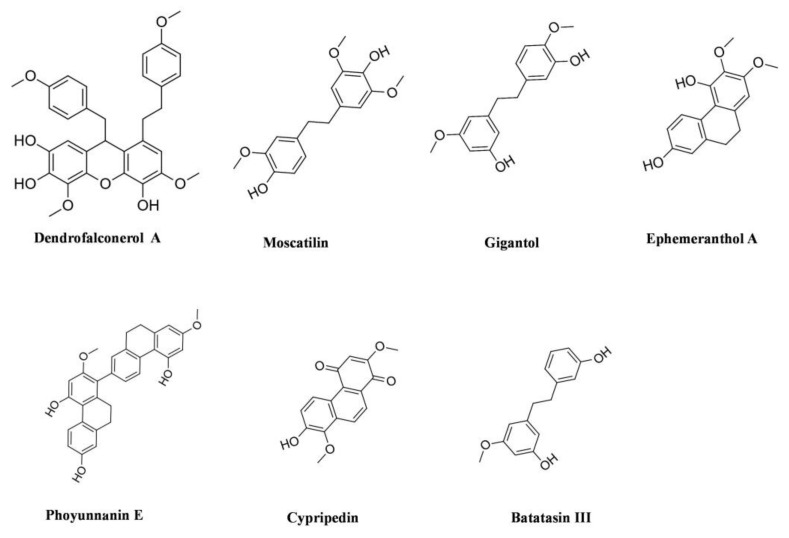
Selected compounds from the Orchidaceae family with anticancer activity.

In vivo studies are the next important step after in vitro and involve testing compounds and assessing the safety of their efficacy on living organisms such as animals, plants or whole cells. The Orchidaceae family is a valuable source of secondary metabolites (selected presented on Figure 2), and despite the limited number of studies meeting our criteria, this is also applicable to the in vivo studies which are presented below.

In the in vivo studies, Su et al. [214] evaluated the antitumour effects of moscatilin, a natural compound isolated from the orchid *Dendrobium moscatum* in the mouse xenograft model. MDA-MB-231 cells were axillary injected into nude mice to establish the mouse model of breast cancer. These data suggested that moscatilin suppresses breast cancer growth and progression in vivo, and therefore can be used as a potential therapeutic agent for the treatment of breast cancer [214]. Sun et al. investigated the possibility of erianin (a natural compound derived from *Dendrobium candidum)*, as a potential therapy in colorectal cancer (CRC). The authors tested the function of erianin on tumor growth in a mouse model by injection of SW480 cells into NOD/SCID mice. These data indicated that erianin inhibited tumor growth via β-catenin in vivo [215]. On the other hand, Zhang et al. investigated the inhibitory effect of *Dendrobium officinale* polysaccharide (DOPA) on human gastric cancer cell SGC-7901 xenografts in nude mice, where the nude mice with SGC-7901 xenografts were randomly divided into model, 5-fluorouracil (5-Fu), low-dose DOPA, middle-dose DOPA, and high-dose DOPA group. DOPA inhibited the growth of SGC-7901 cell xenografts in nude mice. The authors suspect that the mechanism may be related to its increase of serum TNF-α and IL-2 levels, up-regulation of Bax protein expression, and down-regulation of Bcl-2 protein expression [216]. Zhao et al. tested *Dendrobium officinale* extracts (4.8 and 2.4 g/kg) which were administered orally to rats from the gastric carcinogenesis model. Compared to the cancer model group, the high-dose of *Dendrobium officinale* extracts significantly inhibited the rate of carcinogenesis. Further analysis showed that *Dendrobium officinale* extracts regulated DNA damage, oxidative stress, and carcinogenesis-related cytokines, and induced cell apoptosis to prevent gastric cancer [217]. Song et al. noted that dendrobine (an alkaloid isolated from *Dendrobium nobile*) enhanced the chemotoxicity of cisplatin against A549 xenograft tumor female BALB/c mice. Treatment with dendrobine or cisplatin resulted in an obvious reduction of tumour size, whereas combination treatment dramatically decreased the tumor size. Additionally, the authors showed that dendrobine chemo-sensitized A549 cells to cisplatin induced apoptosis through the JNK/p38 pathway in vivo [176]. In turn, Fang et al. investigated if polysaccharides isolated from *Rhizoma pleionis* (PRP) suppress H22 tumor growth in vivo in a model of malignant ascites in BALB/c mice. H22 cells were transplanted into the left abdominal cavity of mice, and then animals were treated either with PRP in saline at various doses (75, 150, and 300 mg/kg) or with cyclophosphamide (CTX) (20 mg/kg) or cyclophosphamide (CTX) (20 mg/kg). The authors revealed that on the tenth day after tumor cell inoculation, the mouse abdominal perimeter and weight in the PRP treatment group were significantly smaller than those in the control group. Collectively, these results demonstrated that PRP has significant antitumour properties in the H22 tumor model [218]. Other studies in xenograft analysis showed that chrysotoxene (phenanthrene derivative that was first isolated from *Dendrobium chrysotoxum*) (20 mg/kg) indicated that it significantly (*p* < 0.01) the inhibited growth of HepG2 cell-induced tumors by regulating the aforementioned apoptotic proteins (Smac, Cytochrome c, Survivin, Bcl-2, Bax, Apaf-1, c-caspase-9, and c-caspase-3), compared with the control group. Finally, the authors suggested that chrysotoxene may be a potential candidate drug for treating patients with hepatoblastoma [219]. Biswas et al. showed that *Bulbophyllum sterile* petroleum ether fraction ameliorates tumour progression in Ehrlich ascites carcinoma model in vivo. The authors revealed that the petroleum fraction of bulbs (PFB) and petroleum fraction of roots (PFR) at the dose of 200 mg/kg reduced the body weight compared to control. Cisplatin, which served as control, was injected on the first day and reduced the increase in body weight as compared to control. Additionally, the results suggested that the active fractions of bulbs and roots possess anticancer activity, likely by inducing apoptosis through the phospho-p53 dependent pathway [137]. A similar antitumour effect in an in vivo model was also shown by Jia et al. These results of antitumour activity demonstrated that the tumor weight of mice in three different dosage groups was significantly lower than that of the model group (*p* < 0.05, *p* < 0.01). Moreover, the authors exhibited that the polysaccharide from the fibrous root of *Bletilla striata* had a significant inhibitory effect on the tumor growth on S_180_ tumor bearing mice. For this reason, the authors suggest that the mechanism of antitumour might be that it could enhance the immune function by regulating the levels of TNF and IL-2 in serum [220]. Kim et al. showed that dendrobine inhibited γ-irradiation-induced migration and invasion of A549 cells by suppressing sulfatase2 (SULF2) expression, thus inhibiting IR-induced signalling. To investigate the inhibitory effects of dendrobine in vivo, a mouse model of IR-induced metastasis, by injecting BALB/c nude mice with γ-irradiated A549 cells via the tail vein, has been established. These results noted that the number of pulmonary metastatic nodules in mice significantly reduced with dendrobine treatment (2 Gy/Dendrobine, 10.87 ± 0.71), by prevention of IR-induced signalling. For this reason, the authors report that this compound may serve as a therapeutic enhancer in non-small cell lung cancer (NSCLC) patients [221].

The studies presented above confirm the enormous anticancer potential of the compounds contained in this family, which makes them potential candidates for future anticancer therapies.

## 9. Conclusions

The review of the literature revealed that orchids have not been equally well studied throughout the world. The largest number of studies refers to Asian orchids, and little is known about the chemical constituents of American and African plants, except the pantropical *Vanilla*.

The literature reports that both extracts and pure compounds extracted from orchids have a strong cytotoxic effect on various cancer cell lines by inducing intrinsic and extrinsic apoptotic pathways. In addition, in vivo studies have shown that pure compounds or extracts can be used as a potential therapeutic agent in anti-cancer therapies. Considering the very low percentage of orchids examined in terms of their secondary metabolites, further analyses are very likely to reveal the existence of numerous new substances suitable for anticancer therapy. 

## Figures and Tables

**Table 1:** Cytotoxic effect and potential mechanism of action of Orchid extracts.

Name of Species	Part of the Plant	Type of Extract	Active Compounds/ Class of Compounds	Cancer Cell Lines	Cytotoxic Effect/Potential Mechanism of Action	Ref
*Acampe praemorsa* (Roxb.) Blatt. & McCann	Leaves	Methanol, ethyl acetate	-	The HeLa and MCF-7	Cytotoxic effect (range 49.27–76.94 µg/mL)	[144]
*Aeridis odarata* Lour.	Leaves	Methanol, ethyl acetate	-	HeLa and MCF-7	Cytotoxic effect (range 26.21–59.06 µg/mL)	[144]
*Eulophia nuda* Lindl.	Tubers	Methanol	-	MCF-7	Cytotoxic effect (1000 µg/mL)	[145]
*Luisia zeylanica* Lindl.	Leaves	Hexane, chloroform, ethyl acetate and methanol	coumarins, flavonoids, glycosides, phenols, saponins, tannins, and terpenoids (2,2-Dimethyl-3-propyloxirane, Hydroperoxide, 1-ethylbutyl, Ethanone, 1-cyclohexyl, Cyclopentanol, 1, methyl, 9,12,15-Octadecatrien-1-ol, 4-Methyl-1,3-dioxane, 5-Oxotetrahydrofuran-2- carboxylic acid, Methyl cis-10-heptadecenoate, (E)9-Octadecenoic acid ethyl ester, Triacontane, Methyl 15-methyl-hexadecanoate€(E)-1-Bis(E)-but-2-enoxy methoxy but-2-ene, Kaempferol 3-glucoside, n-Tridecanoic acid methyl ester, 1, 4-Dimethyl-1,4,6,7-tetrahydroimidazo 4, 5-e 1,4 diazepine-5,8-dione, Phthalic acid, butyl hexyl ester, (Z)-Icos-13-enoic acid, Octadecanoic acid, ethyl ester	MCF-7 and HeLa	Cytotoxic effect (values ranging between 18.36 µg/ml to 67.914 µg/mL)	[146]
*Vanda Tessellata* Hook. Ex G.Don	Roots	Methanol n-hexane and ethyl acetate	flavonoids, tannins, saponins, terpenoids, steroids and alkaloids	Hep-G2 and B16-F10	Cytotoxic effect (range 95.3–145.9 µg/mL)	[147]
*Acampe praemorsa* (Roxb.)	Leaves	Ethanol	-	A549	-	[148]
*Dendrobium officinale* Kimura et. Migo	Whole plant	Ethanol	polysaccharide	MCF-7	(the possible mechanism may be that, extract up-regulates the LC3-II expression, down-regulates the LC3-I expression and p62 expression. In addition, extract inhibits the expression of PI3K and Akt and their phosphorylation, and promotes the expression of PTEN)	[149]
*Eulophia nuda* Lindl.	Tubers	Alcohol, hydro alcoholic and aqueous	-	MCF-7	Cytotoxic effect (285.1 µg/mL)	[150]
*Dendrobium lasianthera* J.J. Sm	Leaves, stem and roots	Chloroform, methanol, and aqueous	terpenoid and phenolic	T47D	Cytotoxic effect (117–628 µg/mL)	[151]
*Arachnis flos-aeris* (L.) Rchb. f.	Leaves, stem and roots	Chloroform, methanol, and aqueous	terpenoid and phenolic	T47D	Cytotoxic effect (139–1436 µg/mL)	[151]
*Phaius mishmensis* Rchb.	Whole plants	n-hexane, chloroform, and ethyl acetate	-	MCF-7, NCI-H460, and SF-268	Cytotoxic effect (1–134 µg/mL)	[152]
*Dendrobium candidum* Wall. ex Lindl.	Whole plant	Methanol	-	SMMC-7721 and BEL-7404	Cytotoxic effect (about 1mg/mL) It is likely that this extract suppressed cell growth by activating mitochondria apoptosis pathway and inducing inhibition of Wnt/β-catenin pathway	[153]
*Dendrobium candidum* Wall. ex Lindl.	Whole plant	-	-	MCF-7	Cytotoxic effect (2 mg/mL) Extract decreased the cell viability of MCF-7 cells by inducing cell cycle arrest at the G2/M phase and regulating the key biomarkers	[154]
*Dendrobium crepidatum* Lindl. & Paxton *and Dendrobium chrysanthum* Wallich ex Lindley	Leaves	Ethanol	-	Dalton’s lymphoma (DL), a murine transplantable T-cell lymphoma	Cytotoxic effect of extract of *D. crepidatum* at 325 μg/mL, while that for the extract of *D. chrysanthum* was 400 μg/mL.	[155]
*Anoectochilus formosanus* Hayata	Whole plants	Methanol	-	SCC-25	Cell viability assay indicates that 1 mg/mL extract inhibited oral cancer SCC-25 cell proliferation by more than 82%	[156]
*Malaxis rheedii* Sw.	Whole plants	Methanol	-	MCF-7 and HeLa	Cytotoxic effect (value of *M. rheedii* on MCF-7 cells was 167.76 μg/mL)	[157]

**Table 2:** Cytotoxic effect and potential mechanism of action of pure compounds or fractions from Orchids.

Name of Species	Part of the Plant	Active Compounds/Isolated	Cancer Cell Line	Cytotoxic Effect/ Mechanism of Action	Ref
*Dendrobium signatum* Rchb. f.	Whole plant	3,4-dihydroxy-3,4-dimethoxybibenzyl, dendrocandin B, dendrocandin I and dendrofalconerol	MDA-231, HepG2 and HT-29	Cytotoxic effect (25.2–137.8 µM)	[180]
*Cymbidium* *finlaysonianum* Lindl	Whole plants (without flowers)	1-(4-Hydroxybenzyl)-4,6-dimethoxy-9,10-dihydrophenanthrene-2,7-Diol, ephemeranthoquinone B, flavanthridin, 2,4-dimethoxyphenanthrene- 3,7-diol, 3,4,6-trimethoxyphenanthrene-2,7-diol, coelonin, lusianthridin, cymbinodin-A	NCI-H187	Cytotoxic effect (3.73 µM)	[181]
*Dendrobium nobile* Lindl.	Stems	Dendroside, isorhamentin-3-*O*-*β*-d-rutinoside, adenosine, 4-methoxy-2,5,9*R*-trihydroxy-9,10-dihydrophenanthrene 2-*O*-*β*-d-glucopyranoside, (7S,8R) dehydrodiconiferyl alcohol 9′-*β*-glucopyranoside, koaburaside, uniperoside, dehydrodiconiferylalcohol-4-*β*-d-glucoside	HeLa, MCF-7 and A549	Cytotoxic effect (from 16.8 to >100 μM)	[182]
*Dendrobium williamsonii* J. Day & Rchb. f.	Whole plants	Aloifol I, moscatilin, moniliformine, balanophonin,	HL-60	Cytotoxic effect (4.48 to 11.04 μM)	[183]
*Liparis nervosa* (Thunb.) Lindl.	Whole plant	Nervosine VII (alkaloid)	HCT116	Nervosine VII simultaneously induced autophagy and apoptosis by activating MAPKs signalling pathway including JNK, ERK1/2 and p38, suppressing the p53 signalling pathway	[184]
*Dendrobium officinale* Kimura et. Migo	Leaves	Denofficin, dendrocandin B, dendrocandin U, 3,4-dihydroxy- 5,4′-dimethoxy bibenzyl, moscatilin, 4,4′-dihydroxy-3,5-dimethoxy Bibenzyl, gigantol	HeLa	Cytotoxic effect (8.0 to 92.4 μM)	[185]
*Liparis nervosa* (Thunb.) Lindl.	The whole plants with roots	Liparisphenanthrenes A, 2,7,2′-trihydroxy- 4,4′,7′-trimethoxy-1,1′-biphenanthrene, 2,2′-dihydroxy- 4,4′,7,7′-tetramethoxy-1,1′-biphenanthrene	HGC-27 and HT-29	Cytotoxic effect (8.21–9.95 μmol/L), (8.53–9.27 μmol/L)	[186]
*Paphiopedilum callosum* (Rchb.f.)	Roots	3′-hydroxy-2,6,5′-trimethoxystilbene, 3′- hydroxy-2,5′-dimethoxystilbene, galangin, 2,3′-dihydroxy-5′-methoxystilbene	MCF-7 and NCI-H187	Cytotoxic effect (62.82–182.48 μM)	[187]
*Dendrobium thyrsiflorum* Rchb.f.	Whole plants	2,7-Dihydroxy-4-methoxyphenanthrene, 2,7-Dihydroxy-4-methoxy-9-fluorenone, 2,3,5-Trihydroxy-4-methoxyphenanthrene, 3,7-Dihydroxy-2,4-dimethoxyphenanthrene, 2,7-Dihydroxy-1,5,6-trimethoxyphenanthrene, 2,5-Dihydroxy-3,4,9-trimethoxyphenanthrene, 2,3,5-Trihydroxy-4-methoxy-9,10-dihydrophenanthrene, Dengibsin, Denchrysan B, 2,5-Dihydroxy-4-methoxy-9,10-dihydrophenanthrene	HL-60 and BEL-7402	Cytotoxic effect (1.61 to 17.25 µM)	[188]
*Dendrobium brymerianum* Rchb.f.	Whole plant	moscatilin, gigantol, lusianthridin, and dendroflorin	H460	Cytotoxic effect (196.7, 23.4, 65.0, and 125.8 μg/mL)	[189]
*Paphiopedilum* *godefroyae* (God.-Leb.) Stein	Roots	2-(3′,5′-dimethoxyphenyl)- 6-hydroxy-5-methoxybenzofuran, 3-hydroxy-2,5′-dimethoxystilbene, 2-(*E*)-2-(3,5-dimethoxyphenyl)- vinyl-phenol, 5,6-dimethoxy-2-(3-hydroxy-5-methoxyphenyl) benzofuran, 2,3′-dihydroxy-5′-methoxystilbene, 2-(5′-hydroxy-3′-methoxyphenyl)-6-hydroxy-5-methoxybenzofuran, 2,3′-dihydroxy-5,5′-dimethoxystilbene, t*rans*-pinostilbene,	NCI-H187	Cytotoxic effect (5.10–168.02 μM)	[190]
*Dendrobium* *findlayanum* Par. & Rchb.f	Stems	(R)-3, α-dihydroxy-4, 4′, 5 -trimethoxybibenzyl., 3, 4-dihydroxy- 3′, 4′, 5- Trimethoxybibenzyl, 3′, 4- dihydroxy-3, 5-dimethoxy bibenzyl, 4, 4′- dihydroxy-3, 3′, 5- trimethoxy bibenzyl, 3, 3′- dihydroxy-5-methoxy bibenzyl, 3, 3′- dihydroxy- 4, 5′-dimethoxy bibenzyl, 4, 4′-dihydroxy-3, 5-dimethoxy bibenzyl	A172, SHSY5Y, and Hela	Cytotoxic effect (1.65–50 μM)	[191]
*Dendrobium falconeri* Hook. f.	Aerial parts	Dendrofalconerol A	H460	Cytotoxic effect (0.5–5 μM)	[192]
*Dendrobium nobile* Lindl.	Stems	dendronbibisline D, dendronbibisline C, dendronbibisline B, dendronbibisline A	HepG2	Cytotoxic effect (1.25, 4.81, 11.99, 19.47 μM)	[193]
*Eulophia macrobulbon* (C.S.P.Parish & Rchb.f.) Hook.f.	Roots	4-methoxy-9,10-dihydro-2,7-phenanthrenediol, 4-methoxy-2,7- phenanthrenediol, 1,5-dimethoxy-2,7-phenanthrenediol, 1,5,7-trimethoxy-2,6-phenanthrenediol, 1-(4-hydroxybenzyl)- 4,8-dimethoxy-2,7-phenanthrenediol	HeLa, CaCo-2 and MCF-7	Cytotoxic effect (17–100 µg/mL)	[194]
*Cremastra appendiculata* (D. Don) Makino	Tubers	Cremaphenanthrene L (1)-P	HCT-116, Hela, MCF-7 and MDA-MB-231	Cytotoxic effect ((1) 15.84–68.81 μM)	[195]
*Dendrobium nobile* Lindl.	Stems	decumbic acid A, decumbic acid B, (−)- decumbic acid, (−)- and (+)-dendrolactone, 4-(3-hydroxyphenyl)-2- butanone, 3-hydroxy-1(3-methoxy-4-hydroxyphenyl)-propan1-one, 3′,4′,5′,-trimethoxycinnamyl acetate	HeLa, MCF-7 and A549	Cytotoxic effect (from 15.3 to 30.0 μM)	[196]
*Dendrobium findlayanum* Par. et Rchb. f	Stems	dendrobine-type alkaloids	HL60, SMMC-7721, A-549 and MCF-7	Cytotoxic effect ( above 40 μM)	[197]
*Arundina graminifolia* (D.Don) Hochr.	Aerial parts	s 7-hydroxy-2,9-dimethoxy-1,4-phenanthrenequinone named arundiquinone, 5,7-dimethoxy-9,10-dihydrophenanthrene-1,2-diol, rac-syringaresinol, ephemeranthoquinone, coelonin	PC12	Cytotoxic effect (about 50 µM)	[198]
*Liparis nervosa* (Thunb. ex A. Murray) Lindl.	Whole plant	nervosine VII, nervosine VIII and nervosine IX	A549, MCF-7and H460	Cytotoxic effect ( >100 mmol/L)	[199]
*Pholidota chinensis* Lind.	Whole plant	polysaccharide	Caco-2	Cytotoxic effect (69.54 μg/mL)	[200]
*Dendrobium plicatile* Lindl.	Aerial parts	2-chloro-3, 4’-dihydroxy-3’,5-dimethoxybibenzyl, 3-methylgiganto (1), 3’-hydroxy-3,4,4’,5-tetramethoxybibenzyl, batatasinIII, moscatilin, erianthridin, coelonin, 2,5-dihydroxy-4-methoxy-9,10-dihydrophenanthrene, lusianthridin, 1,4,7-trihydroxy-2-methoxy9,10-dihydrophenanthrene, emphernathol A, 3,7-dihydroxy-2,4-dimethoxy-9,10-dihydrophenanthrene and calanhydroquinone C three known phenanthrene, 3,7-dihydroxy-2,4-dimethoxy-phenanthrene, nudol and denthyrsinin	MDA-MB231, HepG2 and A549	Cytotoxic effect ((1) 3.41, 3.02, 2.80 µM)	[201]
*Cymbidium faberi* Rolfe	Roots	Coelonin, Shancidin, 1-(4-hydroxybenzyl)-5,7-dimethoxy- phenanthrene-2,6- diol, 5,7- dimethoxyphenanthrene-2,6-diol	SMMC-7721, A549 and MGC80-3	Cytotoxic effect (Shancidin 12.57, 18.21, 11.60 µM)	[165]
*Dendrobium wardianum* Warner	Stems	dendrocandin V, phenanthrenes (denbinobin, 9,10-dihydro-denbinobin, mostatin, loddigesiinols A	HL-60, A-549, SMMC-7721, MCF-7, and SW-480	Cytotoxic effect (2.33–38.48 μM)	[202]
*Dendrobium officinale* Kimura et. Migo	Stems	Fraction polysaccharides	MDA-MB-231, A549 and HepG2	Cytotoxic effect (0.25–3 mg/mL)	[203]
*Dendrobium officinale* Kimura et. Migo	Leaves	polysaccharides	U2OS and Saos-2	Cytotoxic effect (ranged 12.5, 25, 50, 100, and 200 μg/mL ) induced cell apoptosis mediated by the mitochondrial pathway by up-regulating P53, Bax, and Bak expression; down-regulating Bcl-2 and Mcl-1 expression; and increasing Cleaved caspase9/Caspase9, Cleaved caspase3/Caspase3, and Cleaved PARP/PARP ratio	[204]
*Dendrobium offcinale* Lindl.	Stems	Polysaccharide fraction	HepG2	Cytotoxic effect (400 μg/ mL) Fraction decreased the expression level of Bcl-2 and increased that of Bax in HepG2 cells	[205]
*Dendrobium venustum* Teijsm. & Binn.	Whole plant	Phoyunnanin E	H460, H292, and A549	Compound inhibit the motility of lung cancer cells via the suppression of EMT and metastasis-related integrins	[206]
*Dendrobium offcinale* Lindl.	Stems	dendrocandin P1, dendrocandin P2, ephemeranthol A, orchinol, 2, 4, 7-trihydroxy-9, 10-dihydrophenanthrene, confusarin, gigantol and tristin	HL-60 and THP-1	Strongest cytotoxic effect (orchinol values of 11.96 and 8.92 μM)	[207]
*Nervilia concolor* (Blume) Schltr.	Whole plant	Nervisides I–J 3β-O-d-xylopyranosyl-1α,24R,31-trihydroxylcycloartan-28-oic acid, 3β-O-d-xylopyranosyl-31-O-acetyl-1α,24R-dihydroxycycloartan-28-oic acid	K562 and MCF-7	Cytotoxic effect (Both compounds 1 and 2 exerted moderate activity against these two cancer cell lines, with respective values of 20.5 and 20.6 µg/mL for 1 and 40.1 and 90.5 µg/mL for 2	[208]
*Dendrobium aurantiacum var. denneanum* (Kerr) Z.H. Tsi	-	Moscatilin	MG-63, A549, SK-N-SH, HCT116, HeLa, HepG2, Panc-1 and BxPc-3	Cytotoxic effect (25 µM, the strongest effect for pancreatic cells) Compound induced apoptosis of pancreatic cancer cells via reactive oxygen species and the JNK/SAPK pathway	[209]
*Goodyera schlechtendaliana* Reichb.f.	Whole grass	Goodyschle A	SGC-7901 and HepG2	Cytotoxic effect (74.9 and 89.80 µM, respectively)	[210]
*Dendrobium draconis* Rchb.f	Stems	Gigantol	NCI-H460	Cytotoxic effect (above 50 µM)	[211]
*Dendrobium nobile* Lindl.	Stems	nobilin E, dendrocandin V	SGC-7901, K562, A549, BEL-7402, and Hela	Cytotoxic effect (Nobilin E values of 17.30, 10.39, 29.03, 20.13, and 22.19 µM, respectively) and cytotoxic effects against K562 with 28.23 µM for dendrocandin V	[212]
*Dendrobium infundibulum* (Lindl.) Kuntze	Whole plant	Ephemeranthol A	NCI-H460	Cytotoxic effect (100 μM)	[169]
*Cattleya tigrina* A. Rich.	Whole plant	triterpene 24-methylenecycloartanol, gigantol, phocantone	HeLa	Cytotoxic effect (86.43–90.67 µg/mL)	[166]
*Dendrobium draconis* Rchb.f.	Stems	Batatasin III	NCI-H460	Cytotoxic effect (25–100 μM) after 48h. Inhibition of cell proliferation (25–100 μM), migration and invasion by suppressing EMT and FAK/AKT/CDC42 pathway.	[108]
*Dendrobium draconis* Rchb.f.	Stems	Gigantol	NCI-H460	Cytotoxic effect (50 μM). Reduction of anchorage-independent growth and in the survival of the cancer cells. Reduction in the ability of the cancer cells to form tumor spheroids, a critical hallmark of CSCs. Reduction of lung CSCs markers, including CD133 and ALDH1A1. Decrease stemness in the cancer cells by suppressing the activation of protein kinase B (Akt) signal which decreased the cellular levels of pluripotency and self-renewal factors Oct4 and Nanog.	[211]
*Dendrobium draconis* Rchb. f.	Stems	Gigantol	NCI-H460	Cytotoxic effect (50 μM). Attenuation of the EMT process in lung cancer cells. The reduction of AKT activity. Decreased transcription and the stability of Slug. Reduction of *β*-catenin activity and Slug transcription. Enhancing GSK-3*β* ubiquitination of Slug, resulting in decreased Slug levels and thereby suppressing the EMT process	[213]
*Dendrobium venustum* Teijsm. & Binn.	Whole plant	Phoyunnanin E	NCI-H460	Cytotoxic effect (25.7 μM). Induction of apoptosis indicated by condensed and fragmented nuclei with the activation of caspase-3 and -9 and poly (ADP-ribose) polymerase cleavage. Phoyunnanin E mediated apoptosis via a p53- dependent pathway by increasing the accumulation of cellular p53 protein. Depletion of antiapoptotic proteins including MCL1 and Bcl2, upregulation of Bax protein. Reduction in the survival of cells.	[134]
*Dendrobium densiflorum* Lindl.	Whole plant	Cypripedin	NCI-H460	The induction of apoptosis at a concentration of >50 μM with the appearance of morphological changes, including DNA condensation and chromatin fragmentation. Activation of caspase-3 and downregulation of the Bcl-2 and Bcl-xL.	[167]
*Dendrobium infundibulum* Lindl.	Whole plant	Ephemeranthol A	NCI-H460	Cytotoxic effect (>50 μM). Concentration-dependent cell apoptosis. At non-toxic concentrations inhibition of anchorage-independent growth of the cancer cells, as indicated by the decreased colony size and number. Ephemeranthol A also had an inhibitory effect on migration. We further found that ephemeranthol A exerts its antimetastatic effects via inhibition of EMT, as indicated by the marked decrease in N-cadherin, vimentin, and Slug. Furthermore, this compound suppressed the activation of focal adhesion kinase (FAK) and protein kinase B (Akt) proteins, which are key regulators of cell migration. As for the anticancer activity, ephemeranthol A induced apoptosis by decreasing Bcl-2 followed by the activation of caspase 3 and caspase 9.	[169]
*Dendrobium officinale* Kimura et. Migo	Leaves	ViceninII	A549 and H1299	Cytotoxic effect effect (>10 μM). ViceninII targets the TGF-_/Smad and PI3K/Akt/mTOR pathways and inhibit TGF-1-induced EMT phenotypes in lung adenocarcinoma A549 and H1299 cells.	[177]
*Liparis nervosa* (Thunb.) Lindl.	Whole plant	Nervosine VII	HCT116	Cytotoxic effect (11.27 to 33.8 μmol·L^−1^). Apoptosis associated with the activation of an intrinsic pathway by caspase-9, -3 and -7. Autophagy- increase of LC3-II and beclin 1 proteins, and the decrease of p62 protein. Induction autophagy and apoptosis activated by MAPKs signalling pathway including JNK, ERK1/2 and p38, suppressing the p53 signalling pathway.	[184]
*Dendrobium venustum* Teijsm. & Binn.	Whole plant	phoyunnanin E	H460, H292 and A549	Cytotoxic effect (50 to 100 μM) Inhibition of the motility of lung cancer cells via the suppression of EMT and metastasis-related integrins.	[206]
